# Foetal and post-natal exposure of sheep to sewage sludge chemicals disrupts sperm production in adulthood in a subset of animals

**DOI:** 10.1111/j.1365-2605.2011.01234.x

**Published:** 2012-06

**Authors:** M Bellingham, C McKinnell, P A Fowler, M R Amezaga, Z Zhang, S M Rhind, C Cotinot, B Mandon-Pepin, N P Evans, R M Sharpe

**Affiliations:** * Institute of Biodiversity, Animal Health and Comparative Medicine, School of Veterinary Medicine, College of Medical, Veterinary and Life Sciences, University of GlasgowUK; † MRC Centre for Reproductive Health, The Queen's Medical Research Institute, University of EdinburghEdinburgh, UK; ‡ Centre for Reproductive Endocrinology & Medicine, Division of Applied Medicine, Institute of Medical Sciences, University of AberdeenAberdeen, UK; § The James Hutton InstituteCraigiebuckler, Aberdeen, UK; ¶ INRA, UMR1198 Biologie du Développement et ReproductionJouy-en-Josas, France

**Keywords:** developmental exposure, environmental chemicals, Sertoli cell, sheep < animal models, sperm production, testis

## Abstract

Exposure to ubiquitous, environmental chemicals (ECs) has been hypothesized as a cause for declining male reproductive health. Understanding the long-term effects of EC exposure on reproductive health in humans requires animal models and exposure to ‘real life’, environmentally relevant, mixtures during development, a life stage of particular sensitivity to ECs. The aim of this study was to evaluate the effects of in utero and post-natal exposure to environmentally relevant levels of ECs, via sewage sludge application to pasture, on the adult male sheep testis. Hormones, liver concentrations of candidate ECs and Sertoli and germ cell numbers in testes of adult rams that were exposed to ECs in sewage sludge in utero, and until weaning via maternal exposure, and post-weaning via grazing pastures fertilized with sewage sludge, were quantified. Evaluated as a single group, exposure to sludge ECs was without significant effect on most parameters. However, a more detailed study revealed that 5 of 12 sludge-exposed rams exhibited major spermatogenic abnormalities. These consisted of major reductions in germ cell numbers per testis or per Sertoli cell and more Sertoli cell-only tubules, when compared with controls, which did not show any such changes. The sludge-related spermatogenic changes in the five affected animals were significantly different from controls (*p* < 0.001); Sertoli cell number was unaffected. Hormone profiles and liver candidate EC concentrations were not measurably affected by exposure. We conclude that developmental exposure of male sheep to real-world mixtures of ECs can result in major reduction in germ cell numbers, indicative of impaired sperm production, in a proportion of exposed males. The individual-specific effects are presumed to reflect EC effects on a heterogeneous population in which some individuals may be more susceptible to adverse EC effects. Such effects of EC exposure in humans could have adverse consequences for sperm counts and fertility in some exposed males.

## Introduction

It is established that exposure to certain environmental chemicals (ECs) at supra-environmental concentrations can have harmful effects on several physiological systems in animals and potentially in humans (Colborn *et al.*, 1993; Langer *et al.*, 1998; Vine *et al.*, 2000). Exposure to such ECs has been hypothesized to account for the high and/or increasing incidence of male reproductive disorders in humans, such as testicular germ cell cancer, cryptorchidism, hypospadias and some cases of low sperm counts. Collectively, these disorders have been proposed to constitute a testicular dysgenesis syndrome (TDS), with a common origin in foetal life (Toppari *et al.*, 1996; Skakkebaek *et al.*, 2001a, b; Skakkebaek & Jorgensen, 2005). Prenatal and early developmental life stages are particularly sensitive to EC effects, (Rhind, 2005) and exposure during these critical periods may alter programming of the reproductive system so as to affect adult reproductive health and productivity (Rhind *et al.*, 2003).

The TDS has been proposed to result from abnormal function of Sertoli cells (SC) and Leydig cells (LC) in the foetal testis (Skakkebaek *et al.*, 2001a, b; Sharpe & Skakkebaek, 2008). SCs play a crucial role in the development of a functional testis and, thus, in expression of a male phenotype. In the foetal gonad, SCs are the first cells to differentiate in the indifferent gonad, an event which triggers seminiferous cord formation, prevention of germ cell entry into meiosis and differentiation and function of LCs (Mackay, 2000). The latter then secrete testosterone and insulin like factor-3 (INSL3), which drive downstream masculinization events and testicular descent (Hutson *et al.*, 1997; Sharpe, 2001). In adulthood, SCs provide physical and metabolic support for germ cell differentiation, meiosis and transformation into spermatozoa. SCs proliferate in perinatal life, which is critical because SC number determines the number of germ cells that can be supported through spermatogenesis, and thus determines the extent of sperm production in adulthood (Sharpe *et al.*, 2003). Given the fundamental role of SCs in testis development and spermatogenesis, altered SC proliferation or functional development in perinatal life will probably have consequences for adult reproductive function and fertility (Skakkebaek *et al.*, 2001a, b; Sharpe, 2010).

The ECs with endocrine disrupting activity (EDCs) have been postulated to be involved in observed increases in TDS disorders (Skakkebaek *et al.*, 2001a, b). However, much of the evidence linking EDC effects to adverse male reproductive development has involved rodent models exposed to supra-environmental concentrations of single chemicals. Extrapolation of such results to humans is difficult because exposure is normally to multiple ECs at concentrations that are generally lower than the ‘safe’, no observed adverse effect, level (NOAEL) for single chemicals. More recently, male rat studies (Kortenkamp, 2008; Rider *et al.*, 2009) in which ECs are present at around their NOAEL, have shown additive adverse reproductive effects of EC mixtures; however, these EC levels were generally supra-environmental.

The ECs include both inorganic (e.g. heavy metals such as cadmium and lead) and organic compounds (e.g. alkylphenols, phthalates, polychlorinated biphenyls and organochlorine pesticides), many of which have endocrine disrupting potential. ECs exert their physiological effects via a variety of mechanisms (Safe, 1994; Safe & McDougal, 2002; Wilson *et al.*, 2008) and hence, their combined effects can be additive, synergistic or antagonistic (Rajapakse *et al.*, 2002). Furthermore, exposure can occur via inhalation, ingestion or direct contact, all of which are generally ill-defined. Health impacts are also influenced by rates of uptake, metabolism and excretion and exposure prenatally via the placenta or postnatally via breast milk (Rhind, 2005). Investigation of the complex mechanistic interactions that might occur following exposure to EC mixtures is therefore extremely difficult, and presents a major emerging issue for research and risk assessment (Kortenkamp, 2008). To realistically evaluate the impact of EC exposure on human male reproductive health, studies aimed at examining the effects of ‘real-life’ exposure to ‘environmentally relevant’ concentrations of a mixture of chemicals, throughout different developmental life stages are critical. Moreover, the use of outbred, long-lived, model species for such studies will better reflect the exposure scenario in humans in which individual variations in genotype, metabolism, exposure rate and route of exposure may alter the exposure outcome. We used sheep exposed to sewage sludge (bio-solids; a by-product of human domestic, agricultural and industrial waste water treatment), applied to their pasture, to examine real-life EC exposure effects. Sewage sludge contains a complex mix of organic and inorganic pollutants (Giger *et al.*, 1984; Webber & Lesage, 1989; Smith, 1995; Stevens *et al.*, 2003), and is widely used for land remediation and as an agricultural fertilizer, including on land used for grazing by farm animals. When applied to pastures, it results only in modest increases, if any, in environmental (soil) EC concentrations (Rhind *et al.*, 2002, 2010) and therefore provides an ideal model to investigate the effects of ‘real-life’ exposure to complex mixtures of environmental concentrations of chemicals/EDCs. We have previously shown, using this model, that male foetuses exposed to an EC mixture via maternal grazing on sludge-treated pasture, exhibit altered testicular development and hormone production (Paul *et al.*, 2005). Foetal ovarian (Fowler *et al.*, 2008) and neuroendocrine development (Bellingham *et al.*, 2009, 2010), bone density and morphology (Lind *et al.*, 2009) and adult behaviour (Erhard & Rhind, 2004) are also altered. In the present study, we have extended our previous work to determine whether developmental exposure to EC in sewage sludge affects the hormonal or spermatogenic functions of the adult testis.

## Materials and methods

### Ethics statement

All animals used in this study were treated humanely with due consideration to the alleviation of pain, suffering, distress or lasting harm, according to the James Hutton Institute's (formerly the Macaulay Land Use Research Institute) Local Ethical Committee and fully licensed by the United Kingdom's Animals (Scientific Procedures) Act 1986 under Project License authority (60/3356). Project license approval automatically includes a prior ethical committee evaluation and approval process, is legally binding and a legal necessity. All in vivo components of the study were conducted at the James Hutton Institute under this legal framework operating at the highest ethical standards. Therefore, separate ethics approvals from the individual research institutions receiving ex vivo tissue samples (University of Glasgow, University of Aberdeen, INRA and MRC Centre for Reproductive Health) are superseded.

### Animals

Texel ewes were mated with Texel rams at the James Hutton Institute's research station at Hartwood, Scotland. Ewes had been maintained throughout their breeding lives on plots fertilized with either sewage sludge at conventional rates (2.25 tonnes dry matter/ha, twice annually; sludge-exposed) or with inorganic fertilizer containing equivalent amounts of nitrogen (225 kg nitrogen/ha/year; Control), according to protocols described previously (Bellingham *et al.*, 2009). The experimental animals were drawn randomly from a larger group of offspring derived from a flock of ewes (approximately 48 per treatment) with a regular age structure i.e. with similar numbers of ewes aged 2, 3, 4 and 5 years at the time of lambing. Male lambs were reared with their dams on the respective treatment plots, until weaning at approximately 4 months of age, according to conventional management protocols. Following weaning, male lambs were maintained on their respective treatments for a further 3 months; thereafter, control and sludge-exposed animals were maintained as a single flock on conventionally managed untreated pasture, or received pelleted feed and hay when housed for a period of time. No supplementary feeds were given to the dams or the experimental animals at any time during the study i.e. their only dietary source of ECs was the pasture, until at least 7 months of age. Furthermore, there was little or no growth of clover or other oestrogenic plant species in any of the pastures and hence, animals were not exposed to additional phytoestrogens.

### Blood and tissue collection

Control (C) and Sludge-exposed (S) rams were euthanized at 19 months of age by administration of a barbiturate overdose (Euthatal; 500 mg/mL; 30 mL, i.v.; Rhone Merieux, Harlow). Prior to slaughter, rams (*n* = 12 per treatment group) were weighed, scored for body condition (BC; (Russel, 1984; Russel *et al.*, 1969). and blood samples were collected for hormone analysis. The liver was removed and weighed, and tissue samples were taken, wrapped in aluminium foil and stored at −20 °C until analysed later for specific ECs. The heart, prostate, adrenal and thyroid glands were also removed and weighed.

### Testis tissue

Post-mortem, testes were removed and weighed. One testis per animal was taken, and two blocks, each of approximately 2 cm^3^ were cut from separate regions, taking care to avoid the central region in which rete testis tissue predominates. Tissue blocks were fixed for 6 h in Bouin's, and then transferred to 70% ethanol and processed in an automated Leica processor (Leica, Nussloch, Germany) before embedding in paraffin wax for immunohistochemical and stereological analysis. One section per testis was used for evaluation as (with the exception of the rete testis) the tissue is generally homogeneous. Before stereological analysis, two testis tissue sections from each animal were examined and compared, to ensure that the ‘phenotype’ of both testes was comparable; this was confirmed for each animal in the present study.

### Hormone analysis

Serum levels of follicle-stimulating hormone (FSH) and luteinizing hormone (LH) were measured in duplicate samples (0.1–0.2 mL) using radioimmunoassays that have been described and validated previously for sheep (McNeilly *et al.*, 1986); the assay standards used were NIDDK-FSH-RP2 and NIH-LH-S12, and assay sensitivities were 0.1 and 0.2 ng/mL for FSH and LH respectively. Mean intra-assay CV for LH was 7.5%. Serum testosterone (T) concentrations were measured in duplicate after extraction of samples (0.2 mL) with diethyl ether using a modification of a previously described protocol (Sheffield & O'Shaughnessy, 1989). Mean intra- and inter-assay CV were 9.4% and 9.6%, respectively, over three assays, and assay sensitivity averaged 0.015 ng/mL. Serum levels of inhibin A (INHA), the main inhibin type produced by Sertoli cells (SCs) in the male sheep (McNeilly *et al.*, 2002), were measured using a two-site, enzyme-linked immunoassay that uses a capture antibody directed against amino acid sequence 82–114 of the human and ovine βA subunit and a C-specific biotinylated monoclonal antibody raised against a synthetic peptide that corresponds to amino acid sequence 1–32 of the human α-C subunit as the detection antibody (Knight *et al.*, 1998); the limit of detection was 20 pg/mL.

### Tissue chemical analysis

Concentrations of selected ECs in liver samples were determined to provide indices of animal exposure during the study. Tissue was analysed to determine concentrations of the following ECs: Diethylhexyl phthalate (DEHP), selected polychlorinated biphenyls (PCBs) congeners (28, 52, 101, 118, 138, 153, 180), polybrominated diphenyl ether (PBDE) congeners (28, 47, 99, 100, 153, 154, 183) and 16 polycyclic aromatic hydrocarbons (PAHs). Concentrations were determined using gas chromatography linked to mass spectrometry (GC/MS) following sample extraction and preparation, according to protocols previously detailed (Rhind *et al.*, 2002, 2005, 2010). Quality control samples were included with each batch of experimental samples analysed. Limits of detection were 0.01 μg/g for DEHP, 0.02 μg/kg for all PCBs, 0.02 μg/kg for PBDE 28, 47, 99 & 100 and 0.50 μg/kg for PBDE 153, 154 & 183. For PAHs, the limits of detection were 1 μg/kg for all except phenanthrene, fluoranthene, benzo[k]fluoranthene, indenol[1,2,3-cd]pyrene and dibenzo[a,h]anthracene, for which they were 5 μg/kg and pyrene, for which it was 15 μg/kg.

### Basic testicular histology

Prior to immunohistochemical analysis, all testis sections were stained with Harris haematoxylin and eosin (H&E) using standard protocols to examine the basic tissue morphology.

### Immunohistochemistry

To facilitate cell counting, testicular sections were immunostained with Wilms’ tumour gene 1 (WT-1; Santa Cruz Biotechnology, Santa Cruz, CA, USA) and VASA (Abcam, Cambridge, UK) to label SCs and germ cells (GCs), respectively, using standard methods (Scott *et al.*, 2007; McKinnell *et al.*, 2009). Briefly, tissues underwent antigen retrieval by pressure cooking in 0.01 M Citrate buffer, pH 6.0 for 5 mins. Endogenous peroxidase was blocked by incubating slides in 3% (vol/vol) H_2_O_2_ in methanol, and sections were incubated overnight at 4 °C with primary antibody. WT-1 antibody was used at a dilution of 1 : 200 and VASA antibody at 1 : 100. Slides were then washed in TBS and incubated with the appropriate biotinylated secondary antibody, followed by incubation with streptavidin-conjugated horseradish peroxidase (Dako, Ely, UK) and visualization of immunostaining using diaminobenzidine (Liquid DAB+; Dako). Representative sections were photographed using a Provis AX70 microscope (Olympus Optical, London, UK) fitted with a Zeiss AxioCam MRc digital camera (Carl Zeiss Ltd, Welwyn Garden City, UK). Images were compiled using Photoshop CS2 (Adobe Systems Inc, Mountain View, CA, USA).

### Determination of SC and GC numbers

Sections immunostained for WT-1 and VASA, as in the previous section, were used for analysis of SC and GC numbers. Careful examination of sections from two fixed tissue blocks per animal showed that there was no discernible difference in the phenotype between blocks. One section per animal was therefore subjected to stereological analysis using an Axio-Imager microscope (Carl Zeiss Ltd) fitted with a Hitachi HV-C20 camera (Hitachi Denshi Europe, Leeds, UK) and a Prior automatic stage (Prior Scientific Instruments Ltd, Cambridge, UK). Image-Pro 6.2 with Stereologer plug-in software (MagWorldwide, Wokingham, UK) was used to select random fields and to place a counting grid over the tissue. Relative cell volume per testis was first determined (Sharpe *et al.*, 2003). Forty fields per section were counted to obtain a percentage standard error value of <5%. Data were converted to absolute volume per testis by multiplying by testis weight (equivalent to volume), and then converted to cell number per testis after determination of mean cell nuclear diameter and volume (average of 120–150 nuclei) using the Stereologer software nucleator function. The number of spermatogonia, spermatocytes and round spermatids were determined.

### Determination of prevalence of Sertoli cell-only (SCO) tubules

**Figure 1:**
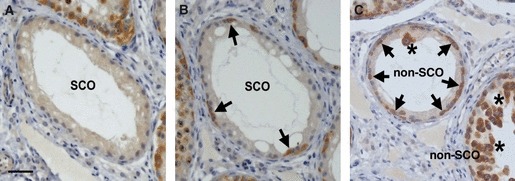
Sertoli cell-only (SCO) tubules in sewage sludge-exposed animals. SCO tubules were defined as those that contained either no VASA positive germ cells (A) or only a few spermatogonia (Sg, arrows) scattered around the basement membrane (B). In contrast, tubules scored as non-SCO (C) either had many Sg scattered around the basement membrane or Sg plus other germ cell types were present (*). Scale bar = 50 μm.

To evaluate the effect of EC exposure on seminiferous tubules, the occurrence of SCO tubules was determined in all animals using sections immunostained with VASA to identify the presence or absence of germ cells. SCO tubules were defined as containing either no germ cells, or a few spermatogonia only distributed sporadically around the basement membrane, and non-SCO tubules were defined as containing many spermatogonia distributed around the entire perimeter of the tubule, or containing any more advanced germ cells (Fig. 1A–C). The stereology system described in the previous section was used to select approximately 80 random fields on one section per testis and all tubules in each field were recorded as being either SCO or non-SCO. The percentage of SCO tubules in each animal was calculated by dividing the number of SCO tubules by the total number of tubules counted (minimum of 250 tubules per section) and multiplying by 100.

### Statistical analyses

For chemical analysis, all data were log-transformed to correct for skewed distribution and analysed using two-way anova. Body and tissue weights and immunoassay data were analysed using Student's *t*-test (*p* < 0.05 significant). Immunohistochemical and stereological data were analysed using one-way anova, except for the incidence of Sertoli cell-only tubules, which used Fisher's exact test. Scatter plots show that the mean and error bars represent the standard error of the mean (SEM).

## Results

### Body and tissue measurements

**Figure 2:**
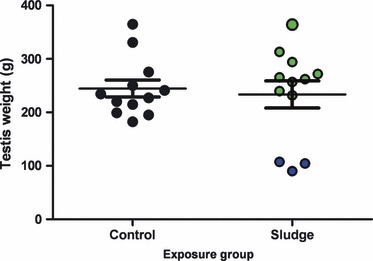
Mean average testis weight in control (C) and sewage sludge-exposed (S) rams. Three rams in the S group (blue circles) had testis weights below the lowest control value and markedly lower than the rest of the S group (s small, *p* < 0.05). Bars represent mean ± SEM.

Mean body weights of C and S rams were not significantly different (C = 77.9 ± 1.0; S = 80.3 ± 1.8 Kg). There was no significant difference in weight between left and right testes in individuals in either control or exposed rams. Although mean testis weights were not significantly different between C and S rams, (C = 245 ± 17; S = 234 ± 27 g) three rams of the sludge-exposed group had markedly smaller testes (<125 g, Fig. 2.) compared with the rest of the exposed group, and one additional ram was noted, at dissection, to have significant accumulation of fluid in the rete testis. There was no significant difference in any of the other gross tissue measurements obtained between control and exposed rams (see Table 1).

**Table 1:** Mean (±SEM) animal and organ weights in control and sludge-exposed rams

Morphological indices	Control (*n* = 12)	Sludge-exposed (*n* = 12)	*p* value
Body weight (kg)	77.9 ± 1.0	80.3 ± 1.8	0.251
Body condition score	2.6 ± 0.07	2.6 ± 0.05	0.694
Liver weight (g)	984 ± 25	1052 ± 48	0.220
Heart weight (g)	358 ± 7.0	378 ± 20.0	0.346
Testis weight (g)	245 ± 17	234 ± 27	0.711
Combined adrenal weight (g)	4.1 ± 0.16	4.1 ± 0.15	1.000
Thyroid weight (g)	5.08 ± .0.25	5.05 ± 0.27	0.929
Prostate weight (g)	18.3 ± 0.8	19.1 ± 1.14	0.587

### Chemical analysis

Although for many chemicals, mean liver concentrations were numerically higher, in C compared with S animals, none of the differences was statistically significant (Table 2). There were no significant correlations between chemical measurements and any physiological parameters measured for animals in either group (data not shown).

**Table 2:** Mean (±SEM) environmental chemical (EC) concentrations (μg per kg of dry liver matter)

Chemical	Chemical	Control	*n*	EC-exposed	*n*	*p* value
Class						
Phthalate	DEHP	0.486 ± 0.108	11	0.707 ± 0.272	10	0.827
PAH	Naphthalene	2.398 ± 0.787	6	6.973 ± 3.250	6	0.383
	Acenaphthalene	1.700 ± 0.433	6	1.878 ± 0.746	6	0.683
	Acenaphthene	3.288 ± 2.129	4	1.893 ± 0.735	4	0.852
	Fluorene	4.110 ± 1.901	4	6.743 ± 4.402	5	0.779
	Phenanthrene	7.467 ± 1.465	8	7.826 ± 2.376	6	0.785
	Anthracene	0.839	1	0.861	1	*
	Fluoranthene		0		0	*
	Pyrene		0		0	*
	Benzo[a]anthracene	8.052 ± 2.017	9	17.88 ± 8.016	7	0.857
	Chrysene	61.55 ± 15.65	12	141.8 ± 56.90	12	0.135
	Benzo[b]fluoranthene	3.566	1	2.800	1	*
	Benzo[k]fluoranthene	2.525 ± 1.009	7	2.144 ± 0.533	7	0.950
	Benzo[a]pyrene	70.63 ± 23.82	12	34.88 ± 8.346	12	0.308
	Indeno[1,2,3-cd]pyrene	13.02	2	3.768	1	*
	Dibenzo[a,h]anthracene		0		0	*
	Benzo[ghl]perylene		0		0	*
	Total PAH	154.2 ± 27.24		216.1 ± 61.70		0.332
PBDE	28	0.011 ± 0.005	11	0.051 ± 0.031	12	0.201
	47	0.316 ± 0.053	11	0.264 ± 0.017	12	0.537
	99	0.028 ± 0.014	3	0.018	1	*
	100		0		0	*
	153		0		0	*
	154	0.448	1	0.360	2	*
	183		0		0	*
	Total PBDE	1.313 ± 0.092		1.203 ± 0.087		0.819
PCB	28	0.019 ± 0.003	7	0.023 ± 0.005	6	0.810
	52		0		2	*
	101	0.016 ± 0.003	4	0.015	2	*
	118	0.014	2	0.017	2	*
	138	0.033 ± 0.013	4	0.232 ± 0.012	5	0.248
	153	0.111 ± 0.025	9	0.080 ± 0.018	11	0.516
	180	0.032 ± 0.014	4	0.022 ± 0.005	5	0.798
	Total PCB	0.177 ± 0.039		0.142 ± 0.028		0.606

DEHP, Diethylhexyl phthalate; PAH, polycyclic aromatic hydrocarbons; PBDE, polybrominated diphenyl ether; PCB, polychlorinated biphenyls.

* Cells show where detectable *n* was <3. Statistical analysis is not appropriate.

### Serum hormone measurements

**Figure 3:**
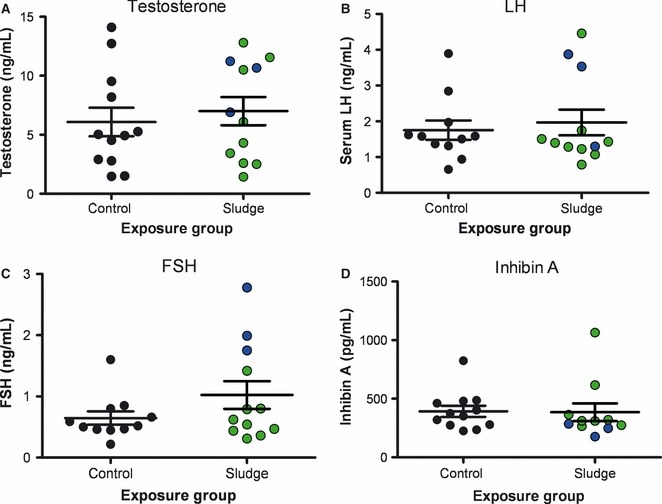
Mean serum concentrations of (A) Testosterone, (B) LH, (C) FSH and (D) INHA in control and sewage sludge-exposed rams. Blue circles = sludge-exposed animals with a low testis weight (as in Fig. 2). Bars represent mean ± SEM.

Concentrations of LH and T immediately prior to euthanasia were not significantly different between C and S rams (Fig. 3A,B). The T:LH ratio was also not significantly different between control and sludge-exposed groups (C = 3.88 ± 0.8; S = 3.76 ± 0.73) and there was no difference in serum T concentration when expressed relative to per gram of testis between C and S groups (C = 12 ± 2; S = 19 ± 5 μg/mL). Mean FSH and Inhibin A levels were also similar in control and sludge-exposed rams (Fig. 3.). However, the three sludge-exposed rams with the significantly smaller testes had high FSH relative to the control range (Fig. 3C).

### Testicular morphology and stereology

**Figure 4:**
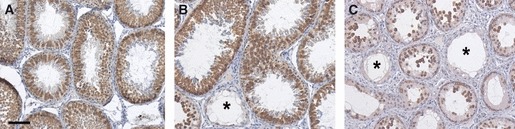
VASA immunostaining of germ cells (GC) in control and sludge-exposed rams. VASA staining shows the phenotype of control animals (A) and two distinct groups of sludge-exposed animals: S1, which had normal GC counts compared with controls (B) and S2, which had statistically significant lower GC counts than controls (C). Note that although S1 animals had normal GC numbers, some also had occasional Sertoli cell-only (SCO) tubules (*), whereas S2 animals all exhibited varying degrees of GC loss and more frequent SCO tubules were evident. Scale bar = 100 μm.

Examination of the H&E stained tissue sections identified several pathological features such as an apparent increase in the number of tubules with dilated lumens, appearance of fewer germ cells in the seminiferous epithelium and appearance of SCO tubules in sections from S animals only (data not shown). To properly quantify these apparent changes, we subsequently examined the morphology of the testis sections using immunohistochemistry. Sections immunostained for VASA were used to evaluate gross testicular morphology and germ cell complement in control and sludge-exposed animals (Fig. 4). The S group exhibited a variable phenotype. In some animals, seminiferous tubules appeared to have a relatively normal germ cell complement and full spermatogenesis, but with a few SCO tubules present in some animals (Fig. 4B). In others, a variable degree of germ cell loss was evident in many tubules, including the complete absence of spermatozoa in some of these animals, and SCO tubules were more frequent (Fig. 4C). SCO tubules were not localized in focal regions, but were interspersed either with tubules in which the germ cell complement was depleted to some degree, or with tubules that appeared relatively normal. We therefore analysed the cellular component of tubules in more detail.

### SC number

**Figure 5:**
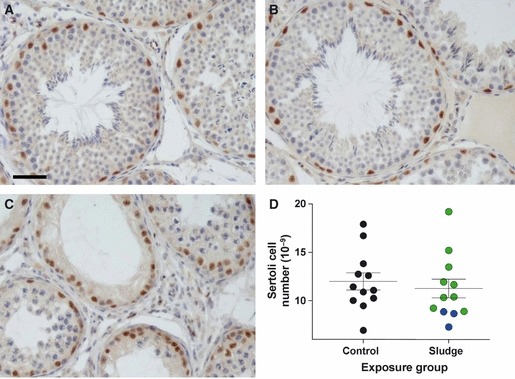
Sertoli cell number in control and sewage sludge-exposed rams. WT-1 immunoexpression, which was unaffected by sludge exposure or germ cell loss, was used to identify Sertoli cells for counting. (A) Control, (B) S1 sludge-exposed, (C) S2 sludge-exposed. Scale bar = 50 μm. (D) Mean Sertoli cell number was not significantly different between control and sludge-exposed animals (S1 + S2). Blue circles = sludge-exposed animals with a low testis weight (as in Fig. 2). Bars represent mean ± SEM.

WT-1 immunoexpression, which was used to label SC nuclei for quantification, was not affected by sludge exposure or by GC depletion (Fig. 5A–C). There was no overall difference in mean SC number per testis between all S animals compared with C (Fig. 5D). SC number was not significantly different relative to testis weight between C and S rams.

### GC numbers and GC volume per testis

**Figure 6:**
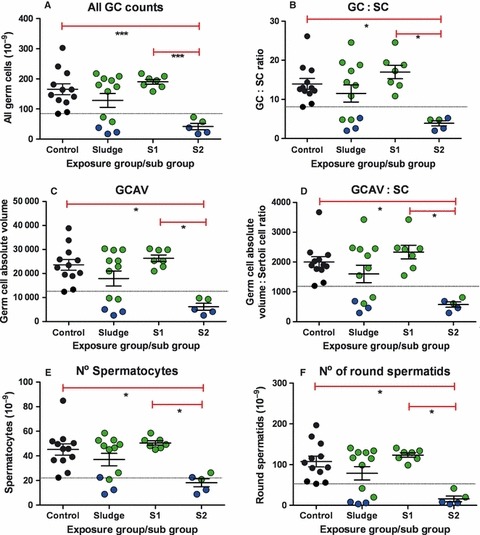
Germ cell numbers/volume in control and sewage sludge-exposed animals. Mean ± SEM data are shown as total germ cell (GC) number per testis (A), GC number per Sertoli cell (SC) (B), the absolute nuclear volume of GC (GCAV) per testis (C), the nuclear volume of GC per Sertoli cell (GCAV : SC) (D) or the number of spermatocytes (E) or round spermatids (F) per testis. Overall, values were not significantly different between control and sludge-exposed rams. However, there were two distinct groups within the sludge-exposed group. S1 were not different to C animals whereas five animals (S2) had GC counts significantly lower than the lowest control (*p < 0.001). The dotted black line represents the lowest control value for each parameter. Red lines show the statistical differences between control and S2 and between S1 and S2. Blue circles = sludge-exposed animals with a low testis weight (as in Fig. 2).

There was a large variation in the ‘normal’ number of total GCs per testis in the control group, which can clearly be seen in Fig. 6A. Numbers of GCs (spermatogonia + spermatocytes + round spermatids) per testis were not significantly different between C and S rams (Fig. 6A). However, 5 of the 12 animals in the S group had total GC counts that were below the lowest control GC value (Fig. 6A). These five animals included the three rams with low testis weights (Fig. 2) and another ram with noticeable fluid in the rete testis, as mentioned in the previous section. Statistical analysis comparing the mean GC counts in the control group to the mean GC counts in the five animals with sub-control GC numbers (termed S2 subgroup) showed that the S2 group had a statistically significant reduction in total GC counts per testis (*p* < 0.001, Fig. 6A). On this basis, data from S animals were therefore subdivided into two distinct groups – S1 comprising animals with mean total GC counts that fell within the control range (84–303 × 10^9^ cells) and S2 comprising the five animals with mean total GC counts that were lower than the control range (17–73 × 10^9^ cells); both subgroups (S1 and S2) were then subjected to further analysis for other histological measurements. Although mean total GC counts in S1 animals were not significantly different from controls, mean total GC counts in S2 animals were statistically significantly reduced (*p* < 0.001) in comparison to both control animals and the S1 animals. GC number per SC was also statistically significantly less in the five S2 animals compared with both the S1 exposed and control animals (*p* < 0.001 Fig. 6B). GC absolute volume (GCAV; calculated for all cell types including spermatozoa) per testis, the ratio of GCAV to SC and numbers of round spermatids and spermatocytes, all exhibited a similar pattern with mean values being statistically significantly reduced in sludge-exposed S2 animals compared with controls (*p* < 0.05, Fig. 6C,5F). However, cell volume per testis for elongated spermatids and spermatozoa were not significantly different in the S2 subgroup compared with controls.

### SCO tubules

There was an increase in the occurrence of SCO tubules in the S group as a whole. Only 1 of 12 animals from the C group had any SCO tubules present, compared with 7 of 12 animals in the S group (Table 3). Of these seven animals, three were from the S1 (*n* = 7) and four from the S2 subgroup (*n* = 5). In the S animals with SCO tubules, a lumen was generally present and the SCs did not appear grossly immature. The four animals with SCO tubules in the S2 subgroup were the same three rams with small testes and the ram with fluid in the rete testis. When the number of animals in S1 and S2 in which SCO tubules were found was compared with the C group, the number in the S2 subgroup was statistically significantly higher than in controls (*p* = 0.027), but S1 was not significantly different from C (Table 3).

**Table 3:** Incidence of Sertoli cell-only (SCO) tubules in the testes of adult males derived from control and sewage sludge-exposed ewes. Significance values are relative to controls

	Number of animals		
		
Exposure group	SCO tubules absent	SCO tubules present (% range)^a^	b*p* value
Control (*N* = 12)	11	1 (1.5%)	
All S exposed (*N* = 12)	5	7 (0.4–21.8%)	0.027
S1 (*N* = 7)	4	3 (0.4–0.7%)	0.117
S2 (*N* = 5)	1	4 (1.0–21.8%)	0.010

a Percentage values in parentheses show the proportion of all seminferous tubules classified as SCO in an individual animal and the range of values between animals.

b Comparison of number of animals with/without SCO tubules (Fisher's exact test).

## Discussion

The results of this study can be interpreted in two very different ways. First, comparison of control and sludge-exposed groups in total for all measured parameters could be interpreted as reassuring evidence that developmental exposure to low level complex EC mixtures is without any statistically significant effect on *any* male reproductive parameter in adulthood in the sheep. The alternative view, which we propose, is that this ‘whole group’ comparison hides adverse testicular changes that are exposure-related, but which are not evident in every sludge-exposed animal. Taking this view, the conclusion reached is entirely opposite to the first view above, namely exposure to ECs present in sewage sludge during prenatal development and early postnatal life is associated with significantly altered sperm production by the adult testis in a significant proportion of sheep. Such a conclusion would support the possibility that TDS and the decline in sperm production in men could be linked with developmental exposure to complex, low level EC mixtures even when the concentration of component chemicals is very low. In this regard, we argue that our results emphasize the relevance of examining the potential impacts of EC exposure using a ‘real-life’ outbred population model, as well as ‘real-world’ EC exposures.

The results of the current study are complex as the effects of EC exposure on aspects of testicular development and function were not the same in all exposed animals. This may be attributable to different EC exposures in individual animals because of uncontrolled factors such as grazing patterns, maternal metabolism or ingestion rates or to differences in individual variation/susceptibility. These are important when examining ‘real-life’ effects of chemical exposure on an outbred population. We initially identified three S animals in which the testes were markedly reduced in weight relative to the other S rams and the controls and another S ram in which there was supranormal fluid accumulated in the rete testis. For reasons of the overall modest size of our study, it could be argued that the occurrence of these three animals in the S group is by chance. We consider that it is more likely to be exposure-related, because in a larger number of control rams (*n* = 26, S. Rhind, unpublished data), no occurrence of testes, this small, has been observed. Similar arguments apply to the animal with rete fluid accumulation, although this was not detectable on the basis of testis size. Furthermore, in a separate study examining sludge exposure during gestation, it was similarly found that approximately 50% of such animals had testis weights (at 140 days gestation) that were below that of the controls (S.M Rhind, unpublished data ). However, what we consider far more definitive is the histological analysis of the testis, which showed grossly abnormal spermatogenesis in the four aforementioned S animals. Moreover, this analysis also identified a further S animal with ‘normal’ sized testes, but with germ cell numbers below the lowest control value. Even in S animals with normal sized testes and normal germ cell counts (i.e. within the control range), some exhibited sporadic abnormalities (SCO tubules). We therefore consider that the logical conclusion from our study is not that there is no effect, but that effects are restricted to a subset of S animals, for reasons that are unidentified.

When assessing adult male reproductive capacity/function, a key measure is sperm producing capacity. Sperm producing capacity is determined by the number of SCs (Orth *et al.*, 1988; Sharpe *et al.*, 2003), and this was unaffected by developmental exposure to ECs in the present study, irrespective of the testicular phenotype (S1 or S2). It was surprising, as in our previous study (Paul *et al.*, 2005), that sewage sludge exposure in utero resulted in reduced SC numbers towards the end of gestation. Moreover, in the present study, sewage sludge exposure extended to birth and beyond into early post-natal life, and hence, ‘reinforcing’ effects on SC number might have been anticipated. Recent evidence from rats shows that even major (approximately 50%) deficits in SC number at birth can be completely compensated for postnatally, if further exposure to insults does not occur (Auharek *et al.*, 2010). While S animals in the current study remained on the treated pasture during the postnatal period, the rates and types (chemical class) of exposure may have differed between pre- and postnatal life, or there may be less vulnerability postnatally. In addition, rams have a longer time period (approximately 4 months) in which SC compensation can occur postnatally, compared with rats (approximately 2 weeks). Alternatively, differences in the animals, weather, maternal grazing patterns or different sludge EC composition, between the present study and that of Paul *et al.* may have resulted in no impact on foetal SC number.

Despite the absence of effect on SC number, the testes of S rams were more likely to contain SCO tubules than controls, and the incidence of SCO tubules was particularly marked in some individuals. SCO tubules are associated with testicular dysgenesis and are commonly found in biopsies from TDS patients (Hoei-Hansen *et al.*, 2003; Nistal *et al.*, 2006; Hutchison *et al.*, 2008). The sporadic nature of their occurrence in affected animals in the present study suggests that it is a local effect and/or may be restricted to specific seminiferous tubules (i.e. a tubule affected along its complete length). However, the underlying cause of the focal absence of germ cells remains unclear. SCO could be indicative of failure of SC maturation (Sharpe *et al.*, 2003; Hutchison *et al.*, 2008), as the role of the SC has to change between perinatal life (proliferation) and adulthood (supporting spermatogenesis). An example of this is the formation of inter-SC tight junctions, which enables seminiferous tubule lumen development (Russell *et al.*, 1989). Deficient tight junction formation can be quite obvious in some TDS patients in which, in focal areas, the SC nuclei appear grossly immature, and a lumen does not form (Hoei-Hansen *et al.*, 2003). However, in the present study, a tubule lumen was generally present in SCO tubules of S animals and the SCs did not appear grossly immature. As SC ‘maturation’ refers to acquisition of many different functions (Fisher *et al.*, 2003), not all of which have been elucidated, it is possible that dysregulation of one or more aspects of this process was focally impaired (presumably during development) in the S2 animals. Future experiments designed to evaluate specific protein markers of SC functional maturation may give a better indication of which, if any, aspects of SC maturation have been affected following EC exposure in this model (Sharpe *et al.*, 2003; Hutchison *et al.*, 2008).

In some sludge-exposed animals (S2), there was an overall reduction in GC numbers per SC. However, this could not be explained by a single factor. For example, it was not because of a high incidence of SCO tubules, as there was no consistent phenotype, but rather a mix of SCO tubules, non-SCO but GC-depleted tubules, tubules with some form of spermatogenic arrest and tubules with apparently normal numbers of GCs in these animals. The relative proportions of these different tubule types differed considerably between individual affected animals, as reflected in the wide variation between S2 animals in the percentage of tubules that were SCO (1–22%). Therefore, the mechanism for reduction in GC per SC in each animal may not be the same. It is highly relevant that this phenotypic mix of tubule types is broadly representative of the phenotypic mix seen in biopsies of the testis in infertile men, (McLachlan *et al.*, 2007) and thus gives general credence to the possibility that such disorders in men could originate because of developmental EC exposure.

The current model of sewage sludge-induced EC mixture exposure and the variation in individual responses to exposure is likely to be more representative of human exposure and responses than that which is found in studies using highly inbred laboratory animals. Furthermore, as reproductive development and spermatogenesis are highly conserved processes, it is logical to expect that similar effects to those found in a subset of sheep in the current study could be expected in some humans that are also chronically exposed to low dose EC mixtures. The consequences for affected humans, however, would probably be far more serious. Humans, unlike rams, do not store spermatozoa, and hence, a reduction in GC number and increase in the number of SCO tubules as found in S2 rams in the present study, would probably have a significant impact on sperm counts in the ejaculate, and thus on fertility (Sharpe, 2010). However, the ability of rams to store spermatozoa would buffer ejaculated-sperm count from the aforementioned deficits. Consistent with this thinking, ejaculated sperm counts were ‘normal’ in sewage sludge-exposed rams (G.B Boe-Hansen *et al*, unpublished data). In addition, as other parameters that may be used to indicate normal reproductive function, i.e. testosterone, INHA, FSH and LH hormonal profiles, and SC number were largely similar in sludge-exposed and control animals, it is clear from this study that such indices cannot be used to indicate whether there is focal depletion of GCs, at least in the ram.

It remains unknown from this study as to which chemical(s) may be responsible for eliciting the effects seen in the testes of sludge-exposed animals as measurement of representative ECs was at the time of exposure, and are probably only relevant for persistent ECs; these did not differ between C and S groups. Nevertheless, similar EC measurement during actual exposure during gestation, in both maternal and foetal compartments, has shown a similar lack of consistent difference between control and sludge-exposed animals (Rhind *et al.*, 2010), re-emphasizing that the sludge exposure model is subtle and ‘real-world’. It must be stressed, however, that sewage sludge contains a mixture of thousands of different chemicals and hence, the measurements reported can only be indicative of potential ‘insults’ and ECs with important effects may not have been measured.

Although our findings must be considered tentative, they indicate that exposure to ECs present in sewage sludge can result in reduced germ cell numbers/sperm production in adulthood. However, the occurrence of such effects is clearly dependent on other (unknown) factors, such as individual genetic make-up and/or metabolism, as not all exposed animals were adversely affected, and those affected varied in the type and severity of effect. Although the results of this study cannot be directly extrapolated to humans, we suggest that the prolonged, low level, multiple EC exposure associated with the sewage sludge exposure is likely to be broadly representative of human EC exposure. As such, the results add to existing knowledge of the potential risks of EDC exposure and highlight potential effects on human reproductive health. Finally, if these sheep studies are a reasonable guide to how humans might respond to environmental pollutant exposure, they suggest that epidemiology-type studies are likely to miss or underestimate the potential impact that EC mixtures might have on individuals. This is because individuals who are susceptible or non-susceptible to a particular level of exposure may be phenotypically indistinguishable. Future studies would benefit from longer, larger scale studies to properly evaluate the effects of EC exposure on an outbred population, where not all animals exhibit the same responses to exposure.
